# *Brucella* spp. distribution, hosting ruminants from Greece, applying various molecular identification techniques

**DOI:** 10.1186/s12917-022-03295-4

**Published:** 2022-05-27

**Authors:** Aristomenis Katsiolis, Dimitrios K. Papadopoulos, Ioannis A. Giantsis, Konstantinos Papageorgiou, Antonis Zdragas, Nektarios D. Giadinis, Evanthia Petridou

**Affiliations:** 1 Department of Microbiology and Infectious Diseases, Aristotle University of Thessaloniki, 54124 Thessaloniki, Greece; 2 Department of Animal Science, Faculty of Agricultural Sciences, University of Western Macedonia, 53100 Florina, Greece; 3 Veterinary Research Institute of Thessaloniki, Hellenic Agricultural Organization DEMETER (former NAGREF), Thessaloniki, Greece; 4 Clinic of Farm Animals, School of Veterinary Medicine, Faculty of Health Sciences, Aristotle University of Thessaloniki, 54124 Thessaloniki, Greece

**Keywords:** Brucellosis, *Brucella* spp., ruminants, PCR, MLVA, BP26

## Abstract

**Background:**

Brucellosis still remains an endemic disease for both livestock and human in Greece, influencing the primary sector and national economy in general. Although farm animals and particularly ruminants constitute the natural hosts of the disease, transmission to humans is not uncommon, thus representing a serious occupational disease as well. Under this prism, knowledge concerning *Brucella* species distribution in ruminants is considered a high priority. There are various molecular methodologies for *Brucella* detection with however differential discriminant capacity. Hence, the aim of this survey was to achieve nationally *Brucella* epidemiology baseline genotyping data at species and subtype level, as well as to evaluate the pros and cons of different molecular techniques utilized for detection of *Brucella* species. Thirty-nine tissue samples from 30 domestic ruminants, which were found positive applying a screening PCR, were tested by four different molecular techniques i.e. sequencing of the 16S rRNA, the BP26 and the OMP31 regions, and the MLVA typing panel 1 assay of minisatellite markers.

**Results:**

Only one haplotype was revealed from the 16S rRNA sequencing analysis, indicating that molecular identification of *Brucella* bacteria based on this marker might be feasible solely up to genus level. BP26 sequencing analysis and MLVA were in complete agreement detecting both *B. melitensis* and *B. abortus.* An interesting exception was observed in 11 samples, of lower quality extracted DNA, in which not all expected MLVA amplicons were produced and identification was based on the remaining ones as well as on BP26. On the contrary OMP31 failed to provide a clear band in any of the examined samples.

**Conclusions:**

The present study reveals the constant circulation of *Brucella* bacteria in ruminants throughout Greece. Further, according to our results, BP26 gene represents a very good alternative to MLVA minisatellite assay, particularly in lower quality DNA samples.

**Supplementary Information:**

The online version contains supplementary material available at 10.1186/s12917-022-03295-4.

## Background

Brucellosis is globally one of the most severe and debilitating zoonoses affecting livestock and humans [1, 2]. Ruminants are also considered the major natural hosts, maintaining the causative agent of the disease in the environment [3–5]. The disease is transmitted to humans either by direct contact with infected animals or by consumption of unpasteurized dairy products [6]. In this context, high prevalence of the disease is accompanied by economic collapse for the stakeholders owing to the produced milk reduction, abortions and forced slaughter. Brucellosis is endemic in countries around the Mediterranean basin, western Asia and parts of Africa as well as Latin America [7] while it constitutes an important occupational disease for breeders, employees in slaughterhouses and veterinarians as well.

The etiological agents of brucellosis are the proteobacteria belonging in the genus *Brucella* (family: Brucellaceae). Twelve species have been described, with *B. abortus* mostly infecting cattle while *B. melitensis* mostly infecting small ruminants. It should be also mentioned that *Brucella melitensis* is responsible for the vast majority of human cases worldwide [8]. In a relevant study in Greece, more than 90% of *Brucella* spp. strains from clinical specimens was identified as *B. melitensis* [9]*.* It should be noted that in Greece, an ongoing vaccination and monitoring program takes place during the last two decades, in an effort to eradicate the disease by slaughtering infected animals and protecting young ones [10–13].

For the effective monitoring of both brucellosis control programs and human disease, it is important to have reliable tests to differentiate vaccine and field strains. Many molecular approaches have been developed to detect vaccine strains [14–17].

More specifically, many PCR based attempts have been conducted to develop an efficient protocol, able to identify *Brucella* genus and distinguish species. At genus specific level, *Brucella* detection molecular tools have been developed targeting various conserved genomic regions, such as the 16S–23S intergenic transcribed [18, 19], the BCSP31 gene [20, 21], the 16S rRNA [22], the perosamine synthetase (per) gene [23], the gene encoding the Omp2a protein antigen [24], the outer membrane proteins (omp2b, omp2a and omp31) [25] and the proteins of the omp25/omp31 family of *Brucella* spp. [26]. Nevertheless, the above assays vary greatly in sensitivity and specificity [27].

PCR based assays targeting species specific level identification are also numerous [28–33], whereas the multi-locus variable-number tandem-repeat analysis (MLVA) assay developed by Le Flèche et al. [30] is considered the gold standard molecular methodology for *Brucella* typing [34]. An appropriate PCR based detection protocol should be able to discriminate vaccine *Brucella* strains (RB51 and Rev1) from pathogenic ones, with many available assays towards this direction [16, 29, 35–37].

Altogether, the main scope of the present study was the investigation of *Brucella* species in ruminants from Greece, at both species and subtype level. Particularly, our objectives were to identify *Brucella* species and subtypes of all the strains, which are very important for epidemiologic surveillance and investigation of outbreaks in brucellosis endemic regions such as Greece [38, 39]. Eventually, in an effort to evaluate the advantages and drawbacks of different molecular techniques utilized for detection and discrimination of *Brucella* species, an additional goal of the study was to evaluate the efficacy and reliability of different *Brucella* identification methodologies.

## Results

### 16S rRNA sequencing and phylogenetic analysis

**Fig. 1:**
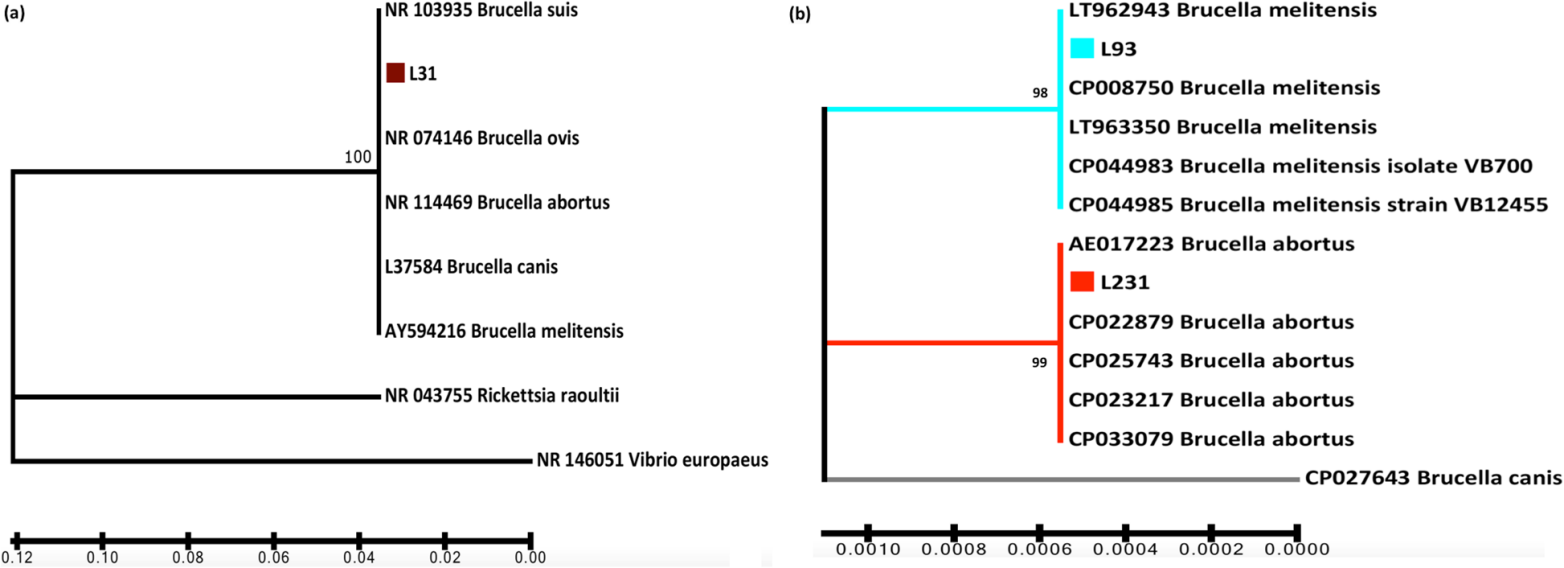
Phylogenetic dendrogram of the examined *Brucella* samples in comparison with haplotypes retrieved from GenBank, based on the 16S rRNA (**a**) and on the BP26 gene (**b**). Haplotypes found in the present study are indicated with a square

Initially all 39 examined samples were subjected to amplification, sequencing and neighbor joining phylogenetic analysis of 16S rRNA. Sequencing results revealed only one identical haplotype in all derived sequences, confirming that all samples belonged to the genus *Brucella* with 100% similarity with other species of *Brucella* (Fig. 1). This haplotype was deposited in the GenBank database and given the accession number OM570553. Those inferences demonstrate that 16S rRNA based molecular identification of *Brucella* bacteria may be feasible solely up to genus level.

### MLVA assay

The panel 1 of MLVA minisatellite markers was applied afterwards in the 39 samples with each PCR being performed at least twice. Firstly, three pairs of primers (Bruce06, Bruce11, Bruce42) were used since they provide an easy to observe differentiation method between the species. Bruce06 amplifies an expected amplicon of 408 bp in case of *Brucella melitensis* and 542 bp in *Brucella abortus*, Bruce11 a product of 257 bp and 383 bp, and Bruce42 539 bp and 289 bp respectively. Afterwards, the pairs of Bruce08 and Bruce12 were used, followed by the remaining three markers i.e. Bruce42, Bruce 43 and Bruce55, which were applied only for samples in which a positive result was observed with at least one of the first five markers, as Bruce42, Bruce 43 and Bruce55 result in an identical amplicon for *Brucella melitensis* and *Brucella abortus*.

The majority of the samples (28 out of the 39) was successfully identified with all the 8 primer pairs of the MLVA assay Panel 1 (Supplementary Material 1). The remaining 11 samples gave the expected amplicon with two or three primer pairs (from the first five markers) and failed to give an amplicon or gave non-specific bands with the rest of the first five primer pairs (Table 1). Interestingly, these samples were of lower DNA quality in terms of purity as measured by the 260/280 absorbance ratio (Table 1).

**Table 1:** Geographic, animal and tissue origin of the examined samples, and *Brucella* identification based on various molecular techniques

Ν	Sample ID	Farm animal species	Originating tissue	Collection site	Screening PCR	MLVA^a^	BP26	OMP31	DNA quality (26/280 ratio)
1	1751	Billy goat	Testicle	Xanthi	*B. melitensis* REV1	*B. melitensis* REV1 (2/8)	*B. melitensis*	–	< 1.2
2	1761	Billy goat	Testicle	Xanthi	*B. melitensis* REV1	*B. melitensis* REV1 (8/8)	*B. melitensis*	–	1.8 – 2.2
3	1771	Billy goat	Testicle	Xanthi	*B. melitensis*	*B. melitensis* (3/8)	*B. melitensisB.*	–	< 1.2
4	3111	Billy goat	Testicle	Xanthi	*B. melitensis* REV1	*B. melitensis* REV1 (8/8)	*B. melitensis*	–	1.8 – 2.2
5	3131	Ram	Testicle	Xanthi	*B. melitensis*	*B. melitensis* (8/8)	*B. melitensis*	–	1.8 – 2.2
6	3141	Billy goat	Testicle	Xanthi	*B. melitensis*	*B. melitensis* (8/8)	*B. melitensis*	–	1.8 – 2.2
7	3171	Ram	Testicle	Xanthi	*B. melitensis* REV1	*B. melitensis* REV1 (8/8)	*B. melitensis*	–	1.8 – 2.2
8	3142	Billy goat	Spleen	Thessaloniki	*B. melitensis*	*B. melitensis* (2/8)	*B. melitensis*	–	< 1.2
9	L81	Bull	Lymph nodes	Thessaloniki	*B. abortus*	*B. abortus* (2/8)	*B. abortus*	–	< 1.2
10	L84	Bull	Lymph nodes	Thessaloniki	*B. abortus*	*B. abortus* (8/8)	*B. abortus*	–	1.8 – 2.2
11	L85	Bull	Lymph nodes	Thessaloniki	*B. abortus*	*B. abortus* (8/8)	*B. abortus*	–	1.8 – 2.2
12	L111	Bull	Lymph nodes	Thessaloniki	*B. abortus*	*B. abortus* (8/8)	*B. abortus*	–	1.8 – 2.2
13	L112	Bull	Lymph nodes	Thessaloniki	*B. abortus*	*B. abortus* (8/8)	*B. abortus*	–	1.8 – 2.2
14	51A	Cow	Embryonic rumen	Thiva	*B. abortus*	*B. abortus* (8/8)	*B. abortus*	–	1.8 – 2.2
15	853	Cow	Embryonic liver	Kalavryta	*B. abortus*	*B. abortus* (8/8)	*B. abortus*	–	1.8 – 2.2
16	3201	Cow	Embryonic rumen	Pella	*B. abortus*	*B. abortus* (8/8)	*B. abortus*	–	1.8 – 2.2
17	L11	Cow	Embryonic rumen	Xanthi	*B. abortus*	*B. abortus* (8/8)	*B. abortus*	–	1.8 – 2.2
18	L12	Cow	Embryonic liver	Xanthi	*B. abortus*	*B. abortus* (8/8)	*B. abortus*	–	1.8 – 2.2
19	L21	Goat	Embryonic rumen	Thiva	*B. melitensis*	*B. melitensis* (8/8)	*B. melitensis*	–	1.8 – 2.2
20	L22	Goat	Embryonic liver	Thiva	*B. melitensis*	*B. melitensis* (3/8)	*B. melitensis*	–	< 1.2
21	L31	Cow	Embryonic liver	Kozani	*B. abortus*	*B. abortus* (8/8)	*B. abortus*	–	1.8 – 2.2
22	L32	Cow	Cotyledonary placenta	Kozani	*B. abortus*	*B. abortus* (8/8)	*B. abortus*	–	1.8 – 2.2
23	L61	Cow	Embryonic rumen	Xanthi	*B. abortus*	*B. abortus* (8/8)	*B. abortus*	–	1.8 – 2.2
24	L62	Cow	Embryonic liver	Xanthi	*B. abortus*	*B. abortus* (8/8)	*B. abortus*	–	1.8 – 2.2
25	L72	Ewe	Embryonic liver	Lesvos island	*B. melitensis*	*B. melitensis* (3/8)	*B. melitensis*	–	< 1.2
26	L93	Ewe	Embryonic liver	Volos	*B. melitensis*	*B. melitensis* (8/8)	*B. melitensis*	–	1.8 – 2.2
27	L121	Cow	Embryonic rumen	Xanthi	*B. abortus*	*B. abortus* (2/8)	*B. abortus*	–	< 1.2
28	L122	Cow	Embryonic liver	Xanthi	*B. abortus*	*B. abortus* (8/8)	*B. abortus*	–	1.8 – 2.2
29	L13	Ewe	Cotyledonary placenta	Larisa	*B. melitensis*	*B. melitensis* (8/8)	*B. melitensis*	–	< 1.2
30	L141	Ewe	Embryonic rumen	Larisa	*B. melitensis*	*B. abortus* (8/8)	*B. abortus*	–	1.8 – 2.2
31	L201	Ewe	Embryonic rumen	Fthiotida	*B. melitensis* REV1	*B. melitensis* REV1 (8/8)	*B. melitensis*	–	1.8 – 2.2
32	L202	Ewe	Embryonic liver	Fthiotida	*B. melitensis*	*B. melitensis* (3/8)	*B. melitensis*	–	< 1.2
33	L221	Cow	Embryonic rumen	Kozani	*B. abortus*	*B. abortus* (3/8)	*B. abortus*	–	< 1.2
34	L222	Cow	Embryonic liver	Kozani	*B. abortus*	*B. abortus* (8/8)	*B. abortus*	–	1.8 – 2.2
35	L223	Cow	Cotyledonary placenta	Kozani	*B. abortus*	*B. abortus* (3/8)	*B. abortus*	–	< 1.2
36	L231	Goat	Embryonic rumen	Magnesia	*B. melitensis*	*B. abortus* (8/8)	*B. abortus*	–	1.8 – 2.2
37	L301	Cow	Embryonic rumen	Trikala	*B. abortus*	*B. abortus* (8/8)	*B. abortus*	–	1.8 – 2.2
38	L312	Cow	Cotyledonary placenta	Florina	*B. abortus*	*B. abortus* (3/8)	*B. abortus*	–	< 1.2
39	L317	Cow	Embryonic liver	Florina	*B. abortus*	*B. abortus* (8/8)	*B. abortus*	–	1.8 – 2.2

^a^Parentheses indicate numbers of markers that worked properly in MLVA identification

### BP26 and OMP31 analysis

Identification based on the BP26 marker was in complete agreement with MLVA results, as only two haplotypes were defined, one assigned to *B. melitensis* and one to *B. abortus*, clearly differentiated from each other and identical with conspecific sequences obtained from GenBank (Fig. 1). These two haplotypes were deposited in the GenBank database and given the accession numbers OM628689 and OM628690, for *B. melitensis* and for *B. abortus* respectively. Nevertheless, this was not always the case regarding the identification with the screening PCR, where there were two samples in which the two methods did not agree (Table 1). Finally, OMP31 marker failed to provide a clear band or sequence in any of the examined samples.

*Brucella* infection summarized results

In total, concerning billy goats all of the examined samples were identified as *Brucella melitensis*, whereas in rams and bulls, only *B. melitensis* and *B. abortus* were identified, respectively. Furthermore, in 3 billy goats and in 1 ram which were PCR positive for vaccine strain Rev1, it was confirmed by MLVA. Regarding females, in 2/3 goats *B. melitensis* was detected, with the remaining on being *B. abortus,* whereas 5/6 ewes hosted *B. melitensis* with again the remaining one being *B. abortus,* and all 17 examined cows hosting *B. abortus.*

## Discussion

In Greece, brucellosis still remains an endemic zoonosis infecting a wide range of animal species with an influence both in public health and in national economy. Although in 1977, a national program against ruminants’ brucellosis was designed and initiated, the disease is not eradicated yet. The generally accepted pathogenic agent of bovine brucellosis is *B. abortus,* wherea *B. melitensis* is only occasionally detected. Respectively, the common pathogenic agent of caprine and ovine brucellosis is *B. melitensis,* whereas *B. abortus* is only rarely found in small ruminants. Thus, small ruminants are considered as the main hosts for *B. melitensis* [5, 40]. Nevertheless, even though Giantzis et al. [41, 42] detected *B. melitensis* in bovine aborted fetuses, they found no *B. abortus* in aborted fetuses from ewes and goats during a five-year period of 1976-1981. The clinical, pathological and epidemiological picture of caprine brucellosis due to *B. melitensis* is similar to *B. abortus* infection in cattle. The dominant strain for human brucellosis is *B. melitensis* [43–46]. On the other hand, Giannakopoulos et al. [47] referred human cases in Western Greece where *B. abortus* was identified as an equally frequent pathogenic agent. On top of that, it is of high importance to investigate whether interspecies transmission of *B. melitensis, B. abortus* and the vaccine strain Rev1 may occur naturally and cause clinical disease in domestic ruminants or may perplex the standard laboratory exams (RBT & CFT). It should be emphasized that control and eradication policies may have to be sometimes readapted to the new data.

In our study, the presence of *B. abortus* was revealed in two such cases, detected from one ewe and one goat. This could be explained by several reasons such as the existence of mixed livestock pastures, the coexistence of sheep and goats in the same shelter with cattle, or occasionally owing to herd movements with infected but non-detected animals. The results suggest cross-species infection of *B. abortus* from cattle to small ruminants raised in close contact [48].

The fact that in four non-vaccinated male small ruminants, the vaccine strain Rev1 was detected (Table 1), possibly indicates that during the vaccine administration in females, errors may have occurred. As a result of the accidental vaccination, the male animals may be characterized as false seropositive during the control program. Finally, due to the aforementioned vaccine administration errors, male animals end up to the slaughterhouse while the farm comes under quarantine for a period of at least 2 to 6 weeks. For these reasons, it is important to improve molecular methods to detect the exact pathogenic agent from samples from alive seropositive animals or in case of slaughter to detect it in a rapid and effective way.

In particular, development and improvement of efficient DNA-based methods is a consistent demand, as well their comparative efficiency. In this context, here we utilized and evaluated four different molecular tools towards this scope. Particularly, apart from the “gold standard” MLVA assay, the 16S rRNA, the OMP31 and the BP26 genes were analyzed. Assays targeting *Brucella* non-coding genomic tandem repeats, such as the MLVA minisatellites [30] or the microsatellite loci proposed earlier [15, 49], have been developed based on the principle of the high genetic homogeneity levels of the different strains, which fail to be distinguished by classic conserved genomic region markers such as the 16S rRNA. Nevertheless, microsatellites, although are excellent markers for genotyping and genetic differentiation studies, on account of hypervariability and high mutation rates may be inadequate to assign the biovar correctly or an isolate at species level. Minisatellites (MLVA) [30] on the contrary, possess a better species identification capability and are moderately variable higher, resulting in higher discriminatory power. On the other hand, gene sequences possess lower mutation rates. In line with Gupta et al. [50] who concluded that for genus-based identification of *Brucella* species, 16S rRNA and 16S-23 rRNA gene are the best target, in our case examination of 16S rRNA produced one identical haplotype in all samples, thus not capable to discriminate the different *Brucella* species. Our results are in agreement with the same study [50] concerning identification based on BP26 gene targets.

Interestingly, BP26 worked better in lower quality samples, where not all minisatellite markers gave product (Table 1). In PCR based methods the quality and purity of *Brucella* spp. DNA is a crucial prerequisite before performing these methods, especially for multiplex PCR methods. Any inhibitor in DNA samples from any source can affect the result of a PCR based method. Sensitivity and specificity of most PCR-based methods are not well established and their real capability for use with clinical samples and hence diagnosis has not been validated [27]. Specifically, for investigation of *Brucella* presence and species identification, samples may originate from slaughterhouses or dead animals, sometimes travelling for days and eventually received at the labs highly degraded. Sequencing of the BP26 gene represents a very good alternative to MLVA minisatellite assay that according to our results constitutes an effective alternative marker in lower quality DNA samples.

## Conclusions

The present research provides clear evidence for the continuing circulation of *Brucella* species in ruminants throughout Greece, a country that still remains therefore endemic in Brucellosis. All but one molecular techniques tested were proved informative and efficient. However our results clearly demonstrate a better performance of BP26 gene marker in lower quality samples, in terms of optical density ratio.

## Methods

### Sample collections

During a period of 3 years (2016-2018), 264 samples were collected from a total of 191 farmed ruminants, originating from farming units located in the mainland of Greece throughout the country. Those samples derived either from positive males to Rose Bengal Test (RBT) and/or Component Fixation Test (CFT) either from aborted fetuses of sheep, goats, and cattle. Thirty-nine (39) out of the 264 tissue samples, which derived from 30 animals, were found PCR positive using the screening Multiplex PCR detection molecular methodology of Garcia-Yoldi et al. [29] for the needs of the Greek Ministry of **Rural Development** [51] and were utilized for the needs of the present study. The 39 samples originated from various organs and tissues from 30 male and female domestic ruminants, i.e. 7 sheep, 7 goats and 16 bovines, including 7 testicles, 1 spleen, 5 lymph nodes, 11 embryonic rumens, 11 embryonic livers, 4 cotyledonary placentas. The above 39 samples were found positive by the Multiplex PCR Assay for *Brucella* spp. [29] in our laboratory [51]. All samples are displayed in detail in Table 1.

### Extraction of genomic DNA from tissue sample

All procedures were performed under the Biosafety Level three (BSL3) guidelines [52]. Tissue samples were initially homogenized within 200μl sterile Phosphate Buffered Saline (PBS) Sigma- Aldrich using a tissue grinder. For homogenization, aseptic processing of all samples was performed by removal of extraneous material and further maceration and chopping into small pieces in PBS. DNA isolation was carried out using the High Pure PCR template preparation kit (Roche, Basel, Switzerland) following the manufacturer instruction with a final elution volume of 80 μl in each sample. The concentration and the quality of the extracted DNA were evaluated in a micro-volume Q5000 UV-Vis Spectrophotometer (Quawell, USA).

### Molecular identification of *Brucella* species

Since our aim was also to evaluate the applicability of different molecular tools for Brucella detection, four different molecular techniques were applied for identification of bacterial species and assignment of *Brucella* positive samples to species level.

Initially the 16S rRNA gene was amplified using the universal for bacteria species primer pair 27F-1492R (Table 2) that may identify the greatest majority of bacteria species at least at genus level. This pair of primers amplifies nearly the complete 16S rRNA. The 27F and 1492R [53] primers corresponded to positions 8–27 and to positions 1492–1513 of *Escherichia coli* 16S rRNA, respectively [56].

**Table 2:** Primers used for *Brucella* identification

Primer Name	Primer sequence (5΄-3΄)	Size of product (bp)^**a**^	Annealing temperature	Reference
Bruce06F	ATGGGATGTGGTAGGGTAATCG	274, 408, 542^**a**^	51^o^C	Le Flèche et al. [30]
Bruce06R	GCGTGACAATCGACTTTTTGTC			
Bruce08F	ATTATTCGCAGGCTCGTGATTC	330, 348, 366^**a**^	51^o^C	Le Flèche et al. [30]
Bruce08R	ACAGAAGGTTTTCCAGCTCGTC			
Bruce11F	CTGTTGATCTGACCTTGCAACC	509, 257, 383^**a**^	51^o^C	Le Flèche et al. [30]
Bruce11R	CCAGACAACAACCTACGTCCTG			
Bruce12F	CGGTAAATCAATTGTCCCATGA	345, 392, 375^**a**^	51^o^C	Le Flèche et al. [30]
Bruce12R	GCCCAAGTTCAACAGGAGTTTC			
Bruce42F	CATCGCCTCAACTATACCGTCA	538, 539, 289^**a**^	51^o^C	Le Flèche et al. [30]
Bruce42R	ACCGCAAAATTTACGCATCG			
Bruce43F	TCTCAAGCCCGATATGGAGAAT	170, 182, 182^**a**^	51^o^C	Le Flèche et al. [30]
Bruce43R	TATTTTCCGCCTGCCCATAAAC			
Bruce45F	ATCCTTGCCTCTCCCTACCAG	187, 151, 151^**a**^	51^o^C	Le Flèche et al. [30]
Bruce45R	CGGGTAAATATCAATGGCTTGG			
Bruce55F	TCAGGCTGTTTCGTCATGTCTT	234, 273, 273^**a**^	51^o^C	Le Flèche et al. [30]
Bruce55R	AATCTGGCGTTCGAGTTGTTCT			
27F	AGAGTTTGATCMTGGCTCAG	1412	50^o^C	Frank et al. [53]
1492R	TACGGY TACCTTGTTACGACTT			
BP26F	GCCCCTGACATAACCCGCTT	1029	58^o^C	Gupta et al. [54]
BP26R	GAGCGTGACATTTGCCGATA			
OMP31F	TGACAGACTTTTTCGCCGAA	720	55^o^C	Vizcaino et al. [55]
OMP31R	TATGGATTGCAGCACCGC			

^a^Products are size-specific for *B. suis*, *B. melitensis* and *B. abortus*, respectively

Positive *Brucella* samples were then subjected to PCRs targeting the BP26 and the OMP31 genes, using the primer pairs BP26F-BP26R [54] and OMP31F-OMP31R [55] (Table 2). BP26 is a conserved gene capable of distinguishing the different *Brucella* species [57]. On the other hand, OMP31 gene is only present in *B. melitensis*. Cloning and sequencing of *B. melitensis* 16M OMP31 [55], a gene coding for a major *Brucella* outer membrane protein, verified that this gene is missing from *B. abortus* strains [58].

Finally, the MLVA typing panel 1 assay of minisatellite markers was applied [30, 59]. For MLVA analysis we worked with the set of primers (Bruce06, 08, 11, 12, 42, 43, 45, 55, Table 2), which amplifies 8 minisatellite markers with a good species identification capability [30]. All PCRs were performed in 20 μl final volumes, containing 0.6 pmol of each forward or reverse primer, 10 μl KAPA 2G Fast Hot Start readymix (Merck, Germany), approximately 50 ng extracted DNA and nuclease free water up to the final volume. Reactions were performed in a FastGene ULTRA Cycler (Nippon Genetics, Japan) and amplification program was as follows: after an initial denaturation at 95^∘^C for 3 min, 35 cycles were performed, of denaturation at 95^∘^C for 30 sec, annealing at 48-60^∘^C (Table 2) for 40 sec, and extension at 72^∘^C for 40-60 sec, depending on the product length (40 sec for products smaller than 1000 bp; 60 sec for products larger than 1000 bp), and final extension at 72∘C for 10 min. The amplified products were examined by electrophoresis in a 1.5% agarose gel stained with ethidium bromide (0.5 mg/ml) and photographed by photo documentation system, or in polyacrylamide gel electrophoresis stained with silver nitrate in cases of very small size products differences separation. To ensure reproducibility, each PCR was performed at least twice. Particularly for 16S rRNA, BP26 and OMP31 regions, successfully amplified products were purified using the NucleoSpin Gel and PCR Clean-up kit (Macherey-Nagel, Germany) following the manufacturer’s recommended protocol and sequenced bidirectionally in an ABI 3730xl automatic sequencer. Sequences produced, were aligned using the software MEGA 7 [60] and compared with conspecific and congeneric ones obtained from the GenBank database in Neighbor Joining phylogenetic trees that were created in the same software applying a bootstrap value of 1000 iterations.

## Supplementary Information

**Additional file 1.**

## Data Availability

The datasets generated and analysed during the current study are available in the NCBI GenBank repository, accession numbers OM628689, OM628690 and OM570553” (https://www.ncbi.nlm.nih.gov/nuccore/OM628689, https://www.ncbi.nlm.nih.gov/nuccore/OM628690, https://www.ncbi.nlm.nih.gov/nuccore/OM570553)

## Abbreviations

MLVA: variable number tandem repeat analysis
RBT: Rose Bengal Test
CFT: Component Fixation Test
