# Supplementary material for: Brucella spp. distribution, hosting ruminants from Greece, applying various molecular identification techniques
Source: BMC Vet Res. 2022 May 27;18:202. doi: 10.1186/s12917-022-03295-4 (PMC9137169; doi:10.1186/s12917-022-03295-4)
Supplement: Supplementary file 1 — Additional file 1. [file 12917_2022_3295_MOESM1_ESM.docx]

Supplementary table 1. MLVA-8 results for each sample investigated, in terms of PCR product length for each primer pair.

| Ν | Sample ID | Bruce06 | Buce08 | Bruce11 | Bruce12 | Bruce42 | Bruce43 | Bruce45 | Bruce55 |
| --- | --- | --- | --- | --- | --- | --- | --- | --- | --- |
| 1 | 1751 | - | 348 | 257 | - | - | - | - | - |
| 2 | 1761 | 408 | 348 | 257 | 392 | 539 | 182 | 151 | 273 |
| 3 | 1771 | 408 | 348 | 257 | - | - | - | - | - |
| 4 | 3111 | 408 | 348 | 257 | 392 | 539 | 182 | 151 | 273 |
| 5 | 3131 | 408 | 348 | 257 | 392 | 539 | 182 | 151 | 273 |
| 6 | 3141 | 408 | 348 | 257 | 392 | 539 | 182 | 151 | 273 |
| 7 | 3171 | 408 | 348 | 257 | 392 | 539 | 182 | 151 | 273 |
| 8 | 1751 | - | 348 | 257 | - | - | - | - | - |
| 9 | L81 | - | 366 | 383 | - | - | - | - | - |
| 10 | L84 | 542 | 366 | 383 | 375 | 289 | 182 | 151 | 273 |
| 11 | L85 | 542 | 366 | 383 | 375 | 289 | 182 | 151 | 273 |
| 12 | L111 | 542 | 366 | 383 | 375 | 289 | 182 | 151 | 273 |
| 13 | L112 | 542 | 366 | 383 | 375 | 289 | 182 | 151 | 273 |
| 14 | 51A | 542 | 366 | 383 | 375 | 289 | 182 | 151 | 273 |
| 15 | 853 | 542 | 366 | 383 | 375 | 289 | 182 | 151 | 273 |
| 16 | 3201 | 542 | 366 | 383 | 375 | 289 | 182 | 151 | 273 |
| 17 | L11 | 542 | 366 | 383 | 375 | 289 | 182 | 151 | 273 |
| 18 | L12 | 542 | 366 | 383 | 375 | 289 | 182 | 151 | 273 |
| 19 | L21 | 408 | 348 | 257 | 392 | 539 | 182 | 151 | 273 |
| 20 | L22 | - | 348 | 257 | 392 | *-* | *-* | - | - |
| 21 | L31 | 542 | 366 | 383 | 375 | 289 | 182 | 151 | 273 |
| 22 | L32 | 542 | 366 | 383 | 375 | 289 | 182 | 151 | 273 |
| 23 | L61 | 542 | 366 | 383 | 375 | 289 | 182 | 151 | 273 |
| 24 | L62 | 542 | 366 | 383 | 375 | 289 | 182 | 151 | 273 |
| 25 | L72 | - | 348 | 257 | 392 | *-* | *-* | - | - |
| 26 | L93 | 408 | 348 | 257 | 392 | 539 | 182 | 151 | 273 |
| 27 | L121 | - | 366 | 383 | - | - | - | - | - |
| 28 | L122 | 542 | 366 | 383 | 375 | 289 | 182 | 151 | 273 |
| 29 | L13 | 408 | 348 | 257 | 392 | 539 | 182 | 151 | 273 |
| 30 | L141 | 542 | 366 | 383 | 375 | 289 | 182 | 151 | 273 |
| 31 | L201 | 408 | 348 | 257 | 392 | 539 | 182 | 151 | 273 |
| 32 | L202 | 408 | 348 | 257 | - | - | - | - | - |
| 33 | L221 | - | 366 | 383 | 375 | *-* | *-* | – | - |
| 34 | L222 | 542 | 366 | 383 | 375 | 289 | 182 | 151 | 273 |
| 35 | L223 | - | 366 | 383 | 375 | *-* | *-* | – | - |
| 36 | L231 | 542 | 366 | 383 | 375 | 289 | 182 | 151 | 273 |
| 37 | L301 | 542 | 366 | 383 | 375 | 289 | 182 | 151 | 273 |
| 38 | L312 | - | - | 383 | 375 | 289 | *-* | - | - |
| 39 | L317 | 542 | 366 | 383 | 375 | 289 | 182 | 151 | 273 |
